# γ-Tocotrienol does not substantially protect DS neurons from hydrogen peroxide-induced oxidative injury

**DOI:** 10.1186/1743-7075-9-1

**Published:** 2012-01-05

**Authors:** Sue-Mian Then, Coral Sanfeliu, Gapor M Top, Wan Zurinah Wan Ngah, Musalmah Mazlan

**Affiliations:** 1 UKM Medical Molecular Biology Institute (UMBI), Universiti Kebangsaan Malaysia (UKM), Kuala Lumpur, Malaysia; 2 Institut d'Investigacions Biomèdiques de Barcelona (IIBB), CSIC-IDIBAPS, Barcelona, Spain; 3 Department of Biochemistry, Faculty of Medicine, Universiti Kebangsaan Malaysia (UKM), Kuala Lumpur, Malaysia; 4 Malaysian Palm Oil Board, Bangi, Selangor, Malaysia

**Keywords:** Apoptosis, Down syndrome, human neurons, oxidative stress, γ-tocotrienol, vitamin E

## Abstract

**Background:**

Down syndrome (DS) neurons are more susceptible to oxidative stress and previous studies have shown that vitamin E was able to reduce oxidative stress and improve DS neurons' viability. Therefore, this study was done to investigate the protective role of γ-tocotrienol (γT3) in DS neurons from hydrogen peroxide (H_2_O_2_) -induced oxidative stress. The pro-apoptosis tendency of γT3 was compared to α-tocopherol (αT) in non-stress condition as well.

**Methods:**

Primary culture of DS and euploid neurons were divided into six groups of treatment: control, H_2_O_2_, γT3 pre-treatment with H_2_O_2_, γT3 only, αT pre-treatment with H_2_O_2 _and αT only. The treatments were assessed by MTS assay and apoptosis assay by single-stranded DNA (ssDNA) apoptosis ELISA assay, Hoechst and Neu-N immunofluorescence staining. The cellular uptake of γT3 and αT was determined by HPLC while protein expressions were determined by Western blot. Comparison between groups was made by the Student's t test, one-way ANOVA and Bonferroni adjustment as well as two-way ANOVA for multiple comparisons.

**Results:**

One day incubation of γT3 was able to reduced apoptosis of DS neurons by 10%, however γT3 was cytotoxic at longer incubation period (14 days) and at concentrations ≥ 100 μM. Pre-treatment of αT and γT3 only attenuate apoptosis and increase cell viability in H_2_O_2_-treated DS and euploid neurons by 10% in which the effects were minimal to maintain most of the DS cells' morphology. γT3 act as a free radical scavenger by reducing ROS generated by H_2_O_2_. In untreated controls, DS neurons showed lower Bcl-2/Bax ratio and p53 expression compared to normal neurons, while cPKC and PKC-δ expressions were higher in DS neurons. On the other hand, pre-treatment of γT3 in H_2_O_2_-treated DS neurons have reduced Bcl-2/Bax ratio, which was not shown in euploid neurons. This suggests that pre-treatment of γT3 did not promote DS cell survival. Meanwhile γT3 and αT treatments without H_2_O_2 _as well as pre-treatment of γT3 and αT induced changes in cPKC and PKC-δ expression in DS neurons suggesting interaction of γT3 and αT with PKC activity.

**Conclusion:**

Our study suggests that γT3 pre-treatment are not sufficient to protect DS neurons from H_2_O_2_-induced oxidative assault, instead induced the apoptosis process.

## Introduction

Vitamin E is a generic term for lipid-soluble, chain breaking antioxidants which consists of four tocopherol isomers (α, β, γ, δ) and four tocotrienol isomers (α, β, γ, δ). The tocopherol and tocotrienol isomers differ in the number and position of methyl substitutions on the chromanol head. Although tocopherols and tocotrienols are closely related chemically, they differ in their biological effectiveness [1]. Studies have shown that vitamin E deficiency impairs cognitive performance in mice subjected to oxidative stress [2]. Meanwhile, one study found that Down syndrome (DS) children have significantly less vitamin E levels than normal children [3]; while another study showed that DS patients with dementia have lower plasma levels of vitamin E than controls without DS [4]. These results suggest that intake of essential nutrients such as folate, vitamin B6, vitamin E, selenium, α-lipoic acid might be important in preventing cognitive deterioration in DS and Alzheimer disease (AD) [5].

However, intervention studies of antioxidant supplementation in DS and AD have not been conclusive. A recent randomized controlled trial on antioxidant supplementation, including vitamin E for DS children did not show any significant difference in developmental outcome after a two-year research period. There was also no significant effect of antioxidant supplementation on the superoxide dismutase and glutathione peroxidase activities, on the superoxide dismutase to glutathione peroxidase ratio and on the urinary isoprostane concentrations [6]. Another recent review that looked at five different studies on antioxidants and cognitive functions revealed that only three studies examining vitamin E and C supplements gave significantly different results-i.e. one study found a positive association with specific cognitive test, while the other two studies showed a link with global cognitive functions [7]. Other double-blind studies reported that vitamin E has no benefit in patients with mild cognitive impairment and Alzheimer's disease [8]. In all these trials, subjects partake high doses of vitamin E (2000 IU or 1500 mg) daily, which is more than the upper tolerable intake level for vitamin E (1500 IU or 1000 mg per day) [9].

Vitamin E mainly function as free radical scavenger, but recent studies showed that tocopherols and tocotrienols have other non-antioxidant roles: α-tocopherol (αT) was shown to modulate signal transduction and gene expression in various cell lines, while tocotrienols possess powerful neuroprotective, anti-inflammatory anti-angiogenic, anti-artherogenic, anti-cancer and cholesterol lowering properties (for a comprehensive review, refer [10]). Vitamin E has been shown to be neuroprotective in various studies: firstly in a landmark study of neurodegeneration of *in vitro *culture of DS neurons [11]; followed by a study that reported that αT was able to attenuate oxidative stress-induced apoptosis in striatal neuron cultures via its free radical scavenger function [12]; while other studies showed that α-tocotrienol protects neurons from glutamate-induced cell death by the c-src activation molecular pathway [13]. However, not many studies have address the possible pro-apoptotic tendency of vitamin E in neurons, especially tocotrienols, which has shown to have greater apoptotic activity towards various cancer cell lines such as mammary tumor cells and prostate tumor cells compared to tocopherols [14,15]. Current studies has shown that tocopherol and vitamin E analogues were able to induced apoptosis in murine C6 glioma cell line and Tet21N neuroblastoma cell line [16,17] but not tocotrienols.

γ-Tocotrienol (γT3) was reported to activate the apoptosis pathway via the mitochondrial death pathway of the Bcl-2 family proteins in pancreatic stellate cells [18], while tocotrienols was shown to be anti-proliferative in mammary epithelial cells by reducing PKCα (Protein Kinase C) activation [19]. Our previous studies in primary rat's astrocytes and cerebellar neuron cultures revealed that high dosage of γT3 was cytotoxic and have a high tendency to induce the expressions of proteins that were involved in the apoptosis pathway such as Bax, p53 and p38 MAPK [20,21]. Another study also showed that high doses of vitamin E and vitamin C enhanced the toxic effect of H_2_O_2 _to cells [22]. Since most trials of vitamin E supplementations utilized high dosage of vitamin E for maximum effects, the concern for the safety of vitamin E supplementation at the molecular and cellular level has yet to be fully addressed. DS cells are known to be highly susceptible to oxidative damage compared to normal cells [23]. Genomic and functional profiling of DS neural progenitor cell line exposed to S100B suggested that dysregulation of chromosome 21 genes led to increased ROS and thereby altered transcriptional regulation of cytoprotective genes in response to oxidative stress [24]. Vitamin E treatment induced neuroinflammatory processes by increasing microglial activation in animals overexpressing S100B, which is involved in the neuropathology of DS and AD [25]. Therefore, the purpose of this study is to further investigate the effects of αT and γT3 in the apoptosis signaling pathway of human DS neurons as a model of oxidative stress susceptible system, while normal human neurons were used as control.

## Materials and methods

### Materials

The Malaysia Palm Oil Board (MPOB) supplied the palm γT3 and αT isomers of 87% and 80% purity respectively, which was isolated as described previously [26]. Culture dishes were from Nunc while antibodies (p53, Bax, Bcl-2, cPKC, PKC-δ, β-actin) were from Santa Cruz Technologies. Reagents for 3-(4,5-dimethylthiazol-2-yl)-2,5-diphenyltetrazolium bromide (MTT) assays were from Promega while single-stranded DNA (ssDNA) Apoptosis ELISA kits were from Chemicon. All other chemicals and reagents were from Sigma unless indicated.

### Primary cortical neuron cultures from normal and DS fetal brain

The cultures were established using human cortical brain tissues obtained from normal euploid and DS legally aborted fetuses at 14-21 weeks of gestation. The permission to use human fetal tissues was obtained from the ethics committee of the Spanish National Research Council (CSIC) (Approval date: August 6th, 2009; ref. no: SAF2009-13093-C02-02). Enriched neuron cultures were prepared as described elsewhere [27].

### Cell culture treatments

Neuron cultures were incubated with varying concentrations of γT3 and αT, with αT as a positive control based on previous studies showing αT having non-toxic and neuroprotective effects on neurons [12,21]. Stock solutions of 0.5 M γT3 and αT (in 100% ethanol) were first resolved overnight in fetal calf serum at 37°C and diluted to 100 times the final concentration with culture media containing 50% ethanol. Final dilution of αT and γT3 in the cell culture contained 0.5% ethanol, which did not significantly affect cell survival (data not shown). All experiments utilized freshly prepared dilutions of H_2_O_2_, αT and γT3.

### Cytotoxicity of γT3

The human DS neuron cultures were incubated with γT3 (1-200 μM) for 24 hours at 37°C. DS neurons were also given γT3 treatment of 7 days and 14 days to determine possible cytotoxicity or protective effects of γT3. Cytotoxicity of γT3 and αT was assessed by propidium iodide (PI) assay. Briefly, for PI assay, the cultures in 96 wells were stained with PI (7 μM) for one hour prior to the end of the incubation period (24 h. 7 days and 14 days). At the end of the incubation period, the fluorescence intensity was determined and expressed relative to cultures treated with 0.2% Triton X-100 (to permeabilize all cells). The fluorescence signal was measured by a fluorescence plate reader (Molecular Devices, USA) at 530-nm excitation ⁄ 645-nm emission to quantify cell membrane damage as described elsewhere [28].

### Detection of Cell Survival

DS and euploid cortical neurons were pre-treated with varying concentrations of αT and γT3 (1-100 μM) for one hour at 37°C, followed by addition of H_2_O_2 _(100 μM) to the cells and a further incubation for 24 hours at 37°C before cell viability and apoptosis were assessed. Cell viability was assessed using MTT assay. Briefly, the cell culture media was loaded with 0.5 mg/mL MTT to detect any decrease in the cell metabolic activity using MTT reduction assay following standard procedures [29]. Meanwhile, the rate of apoptosis was measured using the ssDNA ELISA kit as described previously [20]. In addition, cell viability was also assessed utilizing the PI assay as described above. For cell imaging, DS neuron cultures were stained with of Neu-N (neuron- specific nuclear protein) antibody to confirm the results shown by the MTT and ssDNA ELISA assay. Briefly, cells were fixed with 4% paraformaldehyde before being permeabilized with 0.25% Triton in PBS for 30 mins. The cells were then washed with PBS, followed by incubation with goat serum at room temperature to block unspecific binding site, and incubation with mouse Neu-N antibody (Chemicon, USA) in 1:200 dilution overnight at 4°C. Subsequently, cultures were washed with PBS and incubated with anti-mouse Alexa Fluor 488 (Molecular Probes, The Netherlands) in 1:2000 dilution for 1 hr at room temperature. After washing with PBS, nuclei were counterstained with Hoescht before visualization under fluorescence microscope (Nikon, Japan) at 40× magnification. The intracellular production of ROS was determined using DCFH-DA assay. Non-fluorescent DCFH-DA was permeable to cell membrane and oxidation of hydroperoxides produced fluorescent 2',7'-dichlorofluorescein (DCF), which was detected by fluorescence plate reader at 485 nm excitation/530 nm emission [30].

### Determination of Vitamin E Uptake by HPLC

The uptake of γT3 and αT was analyzed using reverse-phase high performance liquid chromatography (HPLC) Fluorescent EM 330 nm, EX 294 nm detector (Shimadzu, Japan) as described previously [20]. Concentration peaks of the samples were compared with tocotrienol rich fraction (TRF) standard and the concentrations of αT and γT3 uptake in cells were calculated as μM/10^6 ^cells.

### SDS-PAGE and Western Blot

Western blot of DS and euploid (normal) cortical neurons in various treatment groups were used to elucidate the expression of proteins involved in the apoptosis signaling pathway including p53, Bax, Bcl-2, cPKC (for detection of common isoforms PKC-α, PKC-β and PKC-γ) and PKC-δ; while β-actin were used as housekeeping protein and loading control. A maximum protective dosage of 10 μM γT3 and αT was used to test if this concentration could induce apoptosis in DS and euploid neurons. The western blots were performed as previously described.

### Statistical Analysis

Each experiment of cultures in microplates was carried out in triplicate wells with at least three independent cultures. The data were reported as mean ± SD of at least three experiments. Comparison between groups was made by the Student's t test, one-way ANOVA and Bonferroni adjustment as well as two-way ANOVA for multiple comparisons. p < 0.05 was considered as statistically significant for Student t-test whereas p < 0.0001 was considered as statistically significant for multi-factor comparisons.

## Results and discussion

**Figure 1:**
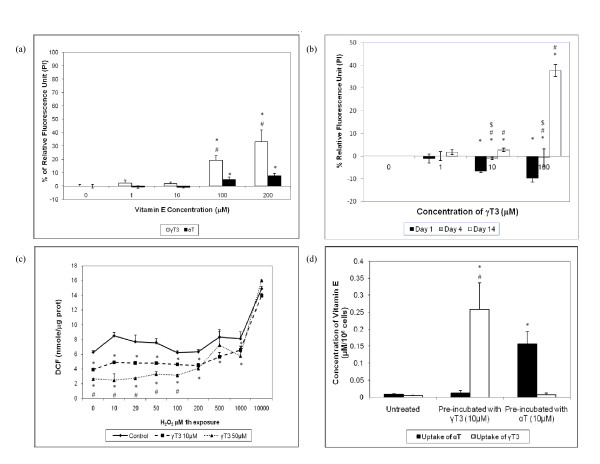
**(a) The effects of α-tocopherol (αT) and γ-tocotrienol (γT3) towards DS neuronal cell death for 14 days *in vitro *as detected by Propidium Iodide (PI) assay**. *Denotes P < 0.05 compared to control while ^# ^denotes P < 0.05 when γT3 was compared to αT at their corresponding concentration. The data are presented as mean ± SD, n = 6. **(b) **The effects of γT3 at various incubation periods (1 day, 4 days and 14 days). *Denotes P < 0.05 compared to the control, while ^**# **^denotes P < 0.05 compared to day 1 and ^**$ **^denotes P < 0.05 compared to day 4. The data are presented as mean ± SD, n = 3. **(c) **The effects of γT3 on ROS generated by H_2_O_2 _in DS neurons with intracellular accumulation of hydroperoxides after one hour exposure to the indicated concentration of H_2_O_2 _measured by DCFH oxidation to DCF. *Denotes P < 0.05 compared to control while ^# ^denotes P < 0.05 compared to 10 μM γT3. The data are presented as mean ± SD, n = 3. **(d) **Uptake of α-tocopherol (αT) and γ-tocotrienol (γT3) in human cortical neurons measured by HPLC. The cellular uptake of γT3 and αT was significantly higher than the untreated control, with the uptake of γT3 significantly higher than that of αT. *Denotes P < 0.05 compared to the control, while # denotes P < 0.05 when γT3 uptake of γT3 incubated cultures were compared with αT uptake of αT incubated cultures. The data are presented as mean ± SD, n = 3.

From the PI assays, 1 μM and 10 μM of γT3 and αT maintained cell viability but did not improve cell survival when it was added to the DS culture for 14 days, γT3 was cytotoxic to DS cortical neurons at concentration ≥ 100 μM compared to αT, with increased apoptosis of 25-35% and 5-8% respectively [Figure 1 (a)]. For neurons incubated with γT3, more apoptotic cells were observed at 50 μM, and at 100 μM almost all of the cells undergo apoptosis. From Figure 1 (b), a short 1 day incubation of γT3 at 10 μM and 100 μM in DS neurons was only able to reduced apoptosis by 10%. The previous landmark study has shown that αT was able to attenuate apoptosis and improve cell viability [11], whereas prolonged incubation time of γT3 up to 14 days increased membrane damage and apoptosis to DS neurons, as detected from the PI assay at a dose dependent manner. Similar to our previous studies, long term incubation of γT3 was shown to be cytotoxic to neurons at high dose (≥ 100 μM) while αT showed minor toxic effects to human neurons as illustrated in Figure 1 (a) and Figure 1 (b)[20,21]. Fluorescence detection of DCF showed that γT3 act as a free radical scavenger by reducing ROS generated by H_2_O_2 _in a dose dependent manner at concentration ≤ 1000 μM of γT3 after one hour H_2_O_2 _exposure [Figure 1 (c)]. The HPLC analysis revealed that the uptake of γT3 was higher compared to the uptake of αT in neurons [Figure 1 (d)]. The absorption of αT had been associated to ATP-binding cassette (ABC) transporter (MDR1) which acts as a cellular exporter of tocopherols to ApoA-I and HDL by ABCA1-dependent and ABCA1-independent processes [31,32]. Hence, this may explain why high doses of αT did not exhibit toxicity to neurons, as αT was effluxed from the cells by ABC transporter 1 (MDR1) to eliminate excess αT. On the other hand, γT3 was not selectively transported by TTP. Thus, excess γT3 was not being effluxed from the cells, leading to the accumulation of γT3 that might exacerbate cell death and contribute to the possible toxicity of γT3 at high dose.

**Figure 2:**
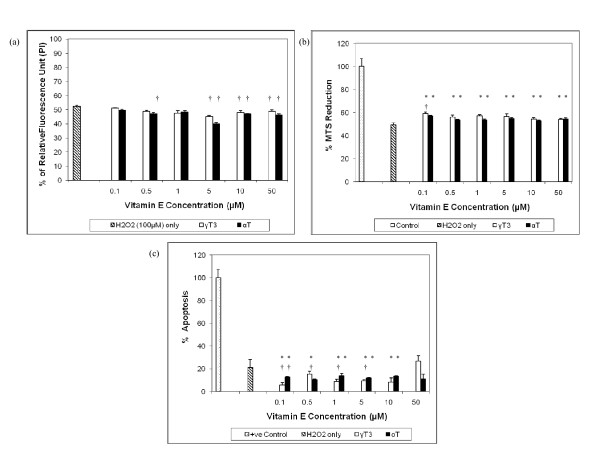
**The effects of α-tocopherol (αT) and γ-tocotrienol (γT3) against H_2_O_2_-induced cell death in human DS neuron cultures, whereby **(a) **the cell death was determined using propidium iodide (PI) assay, **(b) **the cell viability was determined using MTT assay, **(c) **the apoptosis assay was determined using ELISA kits for ssDNA**. The neurons were pre-treated with varying concentrations of αT and γT3 for one hour before the exposure to 100 μM H_2_O_2 _for 24 hours at 37°C. *Denotes P < 0.05 compared to control, † denotes P < 0.0001 compared to H_2_O_2_. The data are presented as mean ± SD, from 3 independent experiments of triplicate wells (n = 9). One-way ANOVA showed that there are significant differences between groups, F_13, 83 _= 14.47, P < 0.001. Meanwhile, two-way ANOVA showed both types of vitamin E isomer and vitamin E concentration are not significant factors contributing to the cell survival and no significant interaction between types of vitamin E isomer and vitamin E concentration (F_5, 71 _= 0.45).

**Figure 3:**
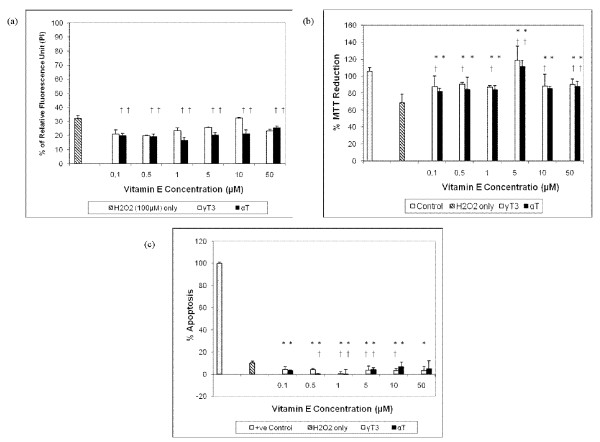
**The effects of α-tocopherol (αT) and γ-tocotrienol (γT3) against H_2_O_2_-induced cell loss in human euploid neuron cultures, whereby **(a) **the cell death was determined using propidium iodide (PI) assay, **(b) **the cell viability was determined using MTT assay, and **(c) **the apoptosis assay was determined using ELISA kits for ssDNA**. The neurons were pre-treated with varying concentrations of αT and γT3 for one hour before the exposure to 100 μM H_2_O_2 _for 24 hours at 37°C. *Denotes P < 0.05 compared to control, † denotes P < 0.0001 compared to H_2_O_2_. The data are presented as mean ± SD, from 3 independent experiments of triplicate wells (n = 9). One-way ANOVA showed that there are significant differences between groups, F_13, 83 _= 9.81, P < 0.001. Two-way ANOVA showed both types of vitamin E isomer and vitamin E concentration are significant factors contributing to the cell survival and no significant interaction between types of vitamin E isomer and vitamin E concentration (F_5, 71 _= 27.15, P < 0.0001).

**Figure 4:**
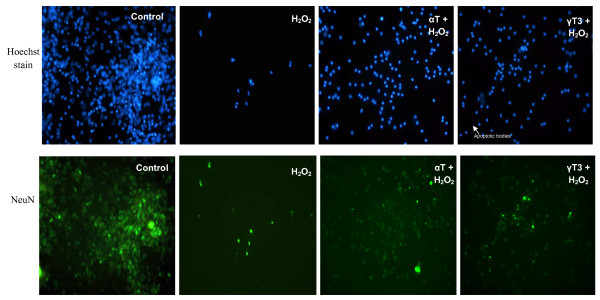
Fluorescence image of DS neurons stained with anti Neu-N antibody in various treatments: control, 100 μM of H_2_O_2 _only, 10 μM of αT or γT3 pre-treatment followed by H_2_O_2 _exposure

In both DS and euploid neurons, the pre-treatment of γT3 and αT at concentration ≤ 50 μM was able to reduce cell death induced by H_2_O_2 _[Figure 2 (a) and Figure 3 (a)] and increase cell viability [Figure 2 (b) and Figure 3 (b)]. Figure 2 (c) and Figure 3 (c) shows that both pre-treatments of up to 10 μM γT3 and 50 μM αT were able to attenuate apoptosis in H_2_O_2_-induced DS and euploid neurons respectively. However, the effectiveness of γT3 and αT pre-treatment was more pronounced in euploid neurons compared to DS neurons, as two-way ANOVA analysis showed that vitamin E isomer type and concentration contributed significantly to cell viability and apoptosis rate in euploid neurons but not in DS neurons. This indicated that the protective effect of the vitamin E in euploid neurons is dose and isomer type dependent, while protective effects of vitamin E in DS neurons is not dependent on dose and isomer type. Figure 4 shows the morphology of H_2_O_2_-treated DS neurons as stained by Neu-N, a marker for differentiated neurons [33] which had undergone apoptosis; pre-treatment with 10 μM of αT or γT3 retained some of the neurons' viability but cell morphologies were not fully maintained. Reduced Neu-N expression in differentiated neurons indicated perturbed cell morphology induced by H_2_O_2 _assault, which pre-treatment of either αT or γT3 were not substantial enough to protect cells from oxidative assault. However, comparatively αT seems to be more protective to the DS neurons than γT3 as more cells were stained with Neu-N, although the staining was dim. This is not surprising since the previous study has shown that *in vitro *DS cortical neuron culture had higher sensitivity to H_2_O_2_-induced oxidative damage compared to euploid neurons [27]. H_2_O_2 _directly induces cellular damage and has been reported to induce parallel apoptosis and autophagy [34], making it more difficult for αT and γT3 to protect the cells especially if the pre-treatment incubation period was short. Since our previous studies have shown that high dose of vitamin E compounds the toxic effect of H_2_O_2_, here we further investigate whether non-lethal doses of high concentration αT and γT3 will further exacerbate the detrimental effects of H_2_O_2 _via the mitochondrial Bcl-2 family pathway or the PKC signalling pathway as both cPKC and PKC-δ are redox sensitive and were reported to be involved in the initiation of apoptosis signalling [35,36].

**Figure 5:**
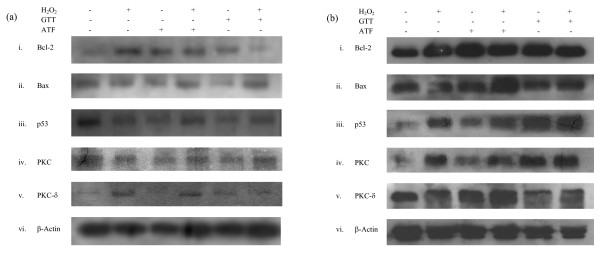
**Bcl-2, Bax, p53, cPKC and PKC-δ were differentially expressed in euploid and DS neurons which were given various treatments in the following fashion: untreated control, incubation of neurons with H_2_O_2 _for 24 hours (H_2_O_2_), incubation of neurons with γT3 (10 μM) for 24 hours (γT3), one hour of γT3 (10 μM) pre-treatment in neurons followed by H_2_O_2 _incubation for 24 hours (γT3 + H_2_O_2_), incubation of neurons with αT (10 μM) for 24 hours (αT) and one hour of αT (10 μM) pre-treatment in neurons, followed by H_2_O_2 _incubation for 24 hours (αT + H_2_O_2_)**. **(a) **Western blot of Bcl-2, Bax, p53, cPKC and PKC in DS neurons; **(b) **Western blot of the same proteins in euploid neurons.

**Figure 6:**
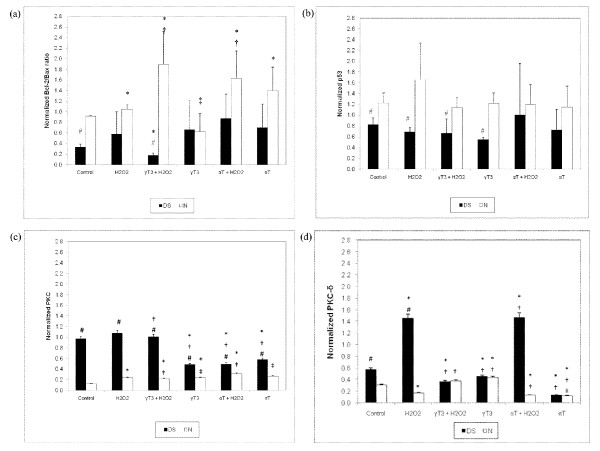
**Densitometric analysis of Bcl-2 and Bax as Bcl-2/Bax ratio in (a), p53 in (b), cPKC in (c) and PKC-δ in (iv)**. * Denotes to P < 0.05 compared to control, † denotes P < 0.05 compared to H_2_O_2_-treated neurons, ‡ denotes P < 0.05 compared to cells pre-incubated with γT3 or αT followed by H_2_O_2 _treatment while # denotes P < 0.05 compares to euploid neurons.

From our investigation, euploid neurons showed a completely different protein expression profile compared to DS neurons. In the control group, DS neurons were shown to have lower Bcl-2/Bax ratio and p53 expression compared to euploid neurons, while cPKC and PKC-δ expressions were higher in DS neurons. Figure 5 (a) (i), Figure 5 (a) (ii) and Figure 6a revealed that for DS neurons, pre-treatment of γT3 followed by H_2_O_2 _has significantly lower Bcl-2/Bax ratio than the controls, whereas other treatment showed changes which were not statistically significant. This result suggests that γT3 does not contribute to the survival of DS neurons under H_2_O_2 _assault via the Bcl-2/Bax heterodimer complex formation. However, the analysis of Western blot for the euploid neurons in Figure 5 (b) (i), Figure 5 (b) (ii) and Figure 6 (a) showed a different picture: Bcl-2/Bax ratio increased significantly in euploid neurons when neurons were pre-treated with either γT3 or αT followed by H_2_O_2 _which suggested that γT3 pre-treatment attenuated apoptosis and improved cell survival of normal euploid neurons. However, p53 expression was not significantly different across various treatments in both DS and euploid neurons, as depicted in Figure 5 (a) (iii), Figure 5 (b) (iii) and Figure 6 (b). Nevertheless, the comparison of p53 expression between DS and euploid neurons showed lower p53 expression in DS neurons for these treatment groups: control, H_2_O_2_, γT3 followed by H_2_O_2 _and γT3 treatments only. Taking it all together, these results show that lower Bcl-2/Bax ratio DS neurons in the untreated groups, was in agreement with the results from previous study which reported that fetal DS neurons had increased Bax and p53 expressions mediated by the transcription factor est-2 when treated with H_2_O_2 _[37]. On the other hand, another study reported that APO-1, caspase-3 and Bcl-2 protein expression levels were unaltered in the fetal DS neurons [38]. A previous study also stated that incubation of αT induced the up-regulation of Bcl-2 as preventive effects from neuronal cell death [39]. Thus, treatment of only αT and γT3 without the presence of H_2_O_2 _in human neuron did not show pro-apoptosis tendency (from the Bcl-2/Bax ratio and p53 expression) compared to rat cerebellar culture as reported previously [21].

Aberrant expression of PKC signalling has been reported in fetal DS post-mortem tissues [40], while DS patients' fibroblast was reported to be hyposensitive to PKC [41]. However, both studies did not specify the location of PKC isoforms involvement. From previous study, cPKC was shown to be activated when exposed to oxidative stress in neuronal death induced by ischemia, hypoxia and exitotoxicity [42], whereas other studies revealed that an increase in PKC-δ expression was needed for glutamate-induced neuronal death [43], Parkinson's disease model [44] and AGE-induced neuronal death [45] as well as H_2_O_2_-induced oxidative stress [46]. Across the various treatment groups, the expression of cPKC was higher in DS neurons compared to euploid neurons. However, the cPKC expression of DS neurons was down-regulated in all other treatment groups (γT3 followed by H_2_O_2_, γT3, αT followed by H_2_O_2_, and αT), as shown in Figure 5 (a) (iv) and Figure 6 (c). From Figure 5 (a) (v) and Figure 6 (d), DS neurons showed a 2-fold increase of PKC-δ expression in H_2_O_2_-treated neurons, suggesting an accumulation of PKC-δ in the cytosol, which signified pro-apoptotic activities in neurons [44] were suppressed by the pre-treatment of γT3, while the pre-treatment of αT did not alter PKC-δ expression. In normal euploid neurons, H_2_O_2 _induced increased cPKC expression [Figure 5 (b) (iv) and Figure 6 (c)] but suppressed PKC-δ [Figure 5 (b) (v) and Figure 6 (d)]. Pre-treatment of γT3 suppressed the cPKC expression but elevated the PKC-δ expression; while the pre-treatment of αT was found to increase the cPKC expression but down-regulated PKC-δ expression [Figure 6 (c) and Figure 6 (d)]. αT has been known to inhibit PKC-α activities [47,48] which was not shown in euploid neurons pre-treated with αT in Figure 5 (b) (iv) and Figure 6 (c). Meanwhile a high concentration of αT (500 μM) has been shown to inhibit PKC-δ activation in AGE-induced neuronal death [45] in which lower dose of αT pretreatment in euploid neurons showed similar result [Figure 5 (b) (v) and Figure 6 (d)]. However, the incubation of only γT3 also showed a decrease in cPKC, similar to a previous study which showed that γT3 suppressed PKC-α expression [19]. This suggests that besides functioning as an antioxidant, γT3 might also play a role in modulating PKC-δ expression as PKC-δ is a redox sensitive molecule.

## Conclusion

This study revealed that in DS neurons, even though γT3 pre-treatment provided initial slight improvement in neuron viability, the protection from both αT and γT3 pre-treatment was not substantial to protect DS neurons from H_2_O_2 _assault. Furthermore, pre-treatment of γT3 would reduce the Bcl-2/Bax ratio that indicates cell survival while αT pre-treatment did not suppress pro-apoptotic PKC-δ expression in the cells. However, in non-oxidative stress condition, αT and γT3 did not exert strong pro-apoptosis tendency in human DS and euploid neurons compared to our previous studies in rat neurons [21]. DS neurons has been shown to have chronic overexpression of S100B, in which oxidation of S100B preferentially induced the neurotrophic processes (which is beneficial for cell survival and differentiation) over neuroinflammation processes. However treatment with antioxidants such as vitamin E interrupts this feedback and leads to increase glial activation and cell death [25]. This may explain the reason why in DS neurons, αT and γT3 at the concentrations used in this study may aggravate cellular damages in a highly susceptible neuronal cell model subjected to oxidative stress. Our present results further underlie the importance of more study to be done on the safety of vitamin E supplementation in neurodegenerative diseases such as DS and AD. Although γT3 act as a free radical scavenger which could quench ROS generated from H_2_O_2_, it may also synergistically induce apoptosis and autophagy through the mitochondrial death pathway, including the Bcl-2 family proteins [49]. Meanwhile, our study also showed that αT has a different mechanism of action compared to γT3, which remains to be further elucidated.

## Competing interests

The authors declare that they have no competing interests.

## Authors' contributions

SMT carried out the most of the experiments, performed the statistical analysis and drafted the manuscript. WZWN and MM played a major role in the experimental procedures of this study and revised the manuscript. CS carried out the cell imaging work and help revised the manuscript. Meanwhile, GMT extracted the vitamin E isomers and tested the purity of the vitamin E isomers. All authors have read and approved the final manuscript.
